# Prenatal diagnosis and management of Apert syndrome in a low-middle income country: Case report

**DOI:** 10.1016/j.ijscr.2024.110134

**Published:** 2024-08-10

**Authors:** Nesrine Souayeh, Asma Marzouk, Hadhami Rouis, Chaouki Mbarki, Hajer Bettaieb

**Affiliations:** a Department of Gynecology and Obstetrics, Ben Arous Regional Hospital, Ben Arous, Tunisia; b Faculty of Medicine, University of Tunis el Manar, Tunis, Tunisia; c Department of Neonatology and Paediatrics, Ben Arous Regional Hospital, Ben Arous, Tunisia

**Keywords:** Apert syndrome, Low-middle income country, Prenatal diagnosis, 3D ultrasound, Case report

## Abstract

**Introduction and importance:**

Apert syndrome is a rare autosomal dominant disorder characterized by craniosynostosis, midface hypoplasia, and syndactyly. Prenatal diagnosis of this condition can be challenging. This case report highlights the importance of recognizing characteristic ultrasound findings for timely diagnosis and genetic counselling.

**Case presentation:**

A 37-year-old, gravida 1, para 0 woman underwent a routine third-trimester ultrasound at 31 weeks gestation. The examination revealed significant hydramnios, bilateral hand syndactyly, foot abnormalities, and an unusual head shape with a prominent forehead, flat occiput, hypertelorism, and low-set ears. These findings raised suspicion for Apert syndrome. Subsequent molecular analysis confirmed a mutation in the FGFR2 gene, confirming the diagnosis. Three-dimensional (3D) ultrasound imaging was utilized to provide the parents with a clearer understanding of the foetal anomalies, aiding in their decision-making process. Given the high risk of impaired intellectual development and the complexity of its management, the pregnancy was terminated at 33 weeks' gestation.

**Clinical discussion:**

This case emphasizes the role of comprehensive prenatal ultrasound in identifying potential foetal anomalies, even in the absence of prior risk factors. Characteristic sonographic findings, such as craniosynostosis, syndactyly, and hydramnios, should raise suspicion for the diagnosis, even in the absence of family history. Molecular confirmation through FGFR2 gene testing is essential for definitive diagnosis and informed genetic counselling.

**Conclusion:**

While Apert syndrome is rare, recognizing its characteristic sonographic features can facilitate timely diagnosis. The use of 3D ultrasound imaging can be invaluable in enhancing parental understanding and facilitating informed decision-making.

## Highlights

• Apert syndrome is a rare genetic condition.
• Prenatal diagnosis might be challenging given the late onset of the ultrasonographic features.
• The use of three-dimensional ultrasound is helpful for both diagnosis and decision-making process.
• Pregnancy termination is usually discussed especially in low-resource countries
• Counselling is mandatory for optimal management.

## 1 Introduction

Apert's syndrome was described for the first time by Wheaton in 1894. In 1906, Dr. Eugene Charles Apert published a summary on nine cases [1]. It is a rare autosomal dominant genetic disorder characterized by coronal craniosynostosis, syndactyly, brachycephaly, midfacial hypoplasia, central nervous system anomalies and variable mental retardation [2,3]. Although there have been cases of Apert syndrome diagnosed prenatally in the literature, many were not discovered until the third trimester, when facial and cranial abnormalities become more apparent [3]. We present a case of a low prior risk couple, in which a strong suspicion of Apert syndrome was made in the third trimester on detailed ultrasound examination and confirmed on prenatal diagnosis. We show how 3D ultrasound was used to provide the parents with a better understanding of the structural defects affecting their baby. This work has been reported in line with the SCARE criteria [4].

## 2 Case presentation

**Fig. 1:**
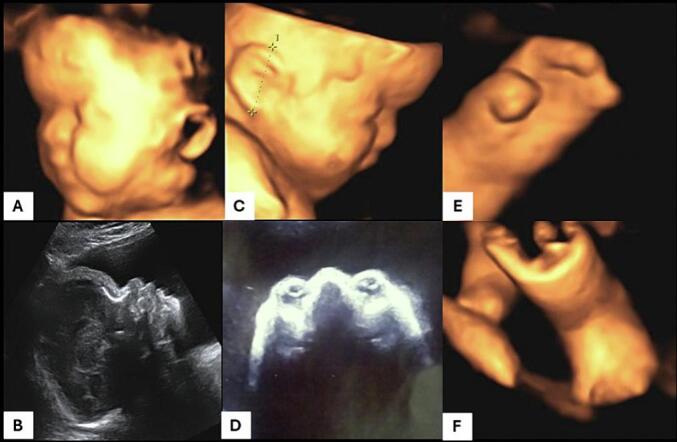
Third-trimester ultrasound findings showing midface hypoplasia with a prominent forehead in 3D (A) and 2D (B), low-set ears (C), hypertelorism, (D) bilateral syndactyly of both hands (mitten hands) appearance) (E), and deformed feet (F).

A 37-year-old gravida 1, para 0, was referred for ultrasonic evaluation at 31 weeks, menstrual age (MA). There was no notion of consanguineous marriage. The couple had a long history of infertility (7 years). The father was 39 years-old with no known high-risk factors for mutations. The current pregnancy was obtained spontaneously after the husband underwent a varicose surgery. The first trimester ultrasound showed a thin nuchal translucency and the presence of the nasal bone. The calculated combined risk of trisomy 21, 13 and 18 was low. The patient did not undergo a second trimester ultrasound. At 31 weeks, a routine ultrasound revealed an important hydramnios. The thorough ultrasound examination performed in our department identified syndactyly of both hands, abnormal feet and an unusual head shape with a prominent forehead and flat occiput, hypertelorism and low inserted ears, resulting in a provisional diagnosis of Apert syndrome being made (Fig. 1). Given the severity of this condition, genetic analysis via amniocentesis was proposed to the couple. Unfortunately, these tests were not available in Tunisia and had to be sent abroad with a considerable processing fee. A sporadic mutation in the Fibroblast Growth Factor Receptor 2 (FGFR2) gene was confirmed on analysis of amniotic fluid (mutation p.Ser252Trp (e.755C>G)). Three-dimensional ultrasound examination was used to provide images of the fetal abnormalities for the parents to aid their understanding of the condition and decision-making process.

**Fig. 2:**
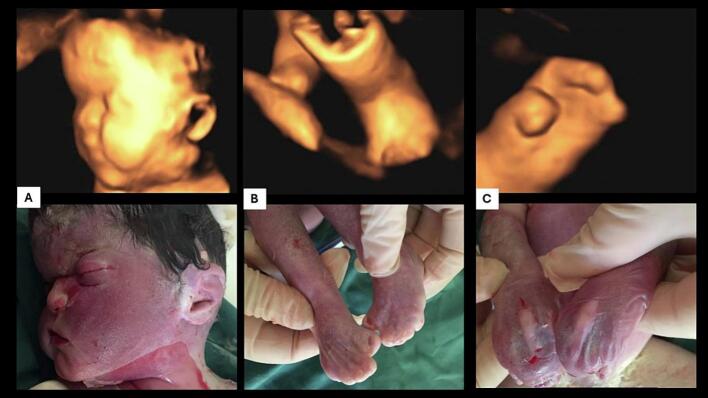
Comparison between 3D ultrasound findings and macroscopic fetal examination showing the same facial dysmorphia (A) and limb abnormalities (B, C).

Parents elected to terminate the pregnancy at 33 weeks' gestation. A female still born was naturally delivered and weighted 1200 g. The macroscopic examination revealed a typical aspect of coronal craniosynostosis and bilateral syndactyly of both hands and feet (Fig. 2).

## 3 Discussion

Apert syndrome accounts for 4.5 % of all patients with craniosynostosis syndromes [2]. The prevalence of Apert syndrome has been estimated at 15 cases per million live births [2]. It is classically characterized by the triad of coronal craniosynostosis, midfacial hypoplasia and symmetric bony syndactyly of the hands and feet [5]. Despite being an autosomal dominant disorder, most cases (>98 %) are sporadic, due to a de-novo mutation in the sperm. The incidence of such mutations increases exponentially with paternal age. It has a significant morbidity, since almost 50 % of affected individuals have a certain degree of mental retardation with IQs ranging from <35 to the normal range [6].

Prenatal diagnosis of Apert syndrome in sporadic cases is challenging because the characteristic sonographic features of craniosynostosis may not be present until the third-trimester [7]. The presence of ocular hypertelorism and proptosis are highly suggestive of Apert syndrome [3]. Midface hypoplasia is classically characterized in Apert syndrome. It results in a deeply depressed nasal bridge, appearing short and wide with a bulbous tip [7]. In our case, the association of hypertelorism and the skull deformation were remarkably suggestive of Apert syndrome. Several reports described abnormal ear morphology in Apert syndrome. In a study published by Farkas, low set ears, and a tendency to disproportion, with widening and small inclination of the longitudinal axis were demonstrated in all subjects [8]. Our case report shows low inserted ears as well.

Fetus with Apert syndrome constantly has abnormalities of the extremities, particularly upper limb syndactyly and careful 2D/3D examination of the extremities confirmed bilateral abnormalities in our case. It is of note that the foot position was abnormal in our case but was not talipes. Syndactyly of the toes is difficult to visualize in utero, but in our case, the feet tended to be short and held in an abnormal position secondary to the syndactyly. It has been shown that 3D ultrasound examination of the skull can reveal premature closure of the coronal suture and a wide metopic suture from the second trimester in Apert syndrome [9]. Moreover, the use of 3D to demonstrate the fetal abnormalities to the parents was found to be particularly useful and aided parental counselling in our case.

Given that the ultrasound features may be not enough the make the diagnosis of Apert syndrome, confirmation refers only to molecular testing. Known mutations that cause the syndrome (found in 98–99 % of cases) are two recurrent missense mutations of the fibroblast growth factor receptor 2 gene (FGFR2) involving two adjacent amino acids (S252W and P253R) [10]. In our case, a heterozygote mutation S252W C>G was detected in the amniotic fluid.

If parents decide to carry on the pregnancy to term, neonates may require management of their airway in the early life. Surgery such as craniofacial disjunction or shunting to reduce intracranial pressure in early childhood has been shown to reduce the incidence of developmental delay [6].

Majority of individuals will require multiple surgeries to correct their syndactyly. In addition, surgeries may be performed later in life to increase intracranial volume to improve neurological outcome or for cosmetic reasons [5].

## 4 Patient's perspective

The couple felt that the sonographic images provided enabled them to understand the severe nature of the condition and make an informed decision.

## 5 Conclusion

Apert syndrome should be diagnosed in the second trimester. This condition may be suspected in low-risk patients when fetal examination reveals abnormal skull shape or contour, especially when 3D imaging is used. Diagnosis confirmation is based on molecular analysis of amniotic fluid. Three-dimensional ultrasound can be used to show limbs and facial abnormalities to the future parents.

## Ethical approval

Ethical approval for this study (Ethical Committee N° 11/2024) was provided by the local committee of our institution on 11 June 2024.

## Funding

This research did not receive any specific grant from funding agencies in the public, commercial, or not-for-profit sectors.

## Author contribution

Drafting the article: Nesrine Souayeh, Hajer Bettaieb Asma Marzouk

Acquisition of data: Hadhami Rouis, Asma Marzouk

Revising the article: Nesrine Souayeh, Hajer Bettaieb , Chaouki Mbarki

All the authors have read and agreed to the final manuscript.

## Guarantor

Nesrine Souayeh.

## Research registration number

Not applicable.

## Conflict of interest statement

Authors declared they have no conflicts of interest.
